# An algorithm for the beat-to-beat assessment of cardiac mechanics during sleep on Earth and in microgravity from the seismocardiogram

**DOI:** 10.1038/s41598-017-15829-0

**Published:** 2017-11-15

**Authors:** Marco Di Rienzo, Emanuele Vaini, Prospero Lombardi

**Affiliations:** Dept. of Biomedical Technology, Fondazione Don Carlo Gnocchi, ONLUS, Milano, Italy

## Abstract

Seismocardiogram, SCG, is the measure of precordial vibrations produced by the beating heart, from which cardiac mechanics may be explored on a beat-to-beat basis. We recently collected a large amount of SCG data (>69 recording hours) from an astronaut to investigate cardiac mechanics during sleep aboard the International Space Station and on Earth. SCG sleep recordings are characterized by a prolonged duration and wide heart rate swings, thus a specific algorithm was developed for their analysis. In this article we describe the new algorithm and its performance. The algorithm is composed of three parts: 1) artifacts removal, 2) identification in each SCG waveform of four fiducial points associated with the opening and closure of the aortic and mitral valves, 3) beat-to-beat computation of indexes of cardiac mechanics from the SCG fiducial points. The algorithm was tested on two sleep recordings and yielded the identification of the fiducial points in more than 36,000 beats with a precision, quantified by the Positive Predictive Value, ≥99.2%. These positive findings provide the first evidence that cardiac mechanics may be explored by the automatic analysis of SCG long-lasting recordings, taken out of the laboratory setting, and in presence of significant heart rate modulations.

## Introduction

The assessment of cardiac mechanics is essential for the full evaluation of the heart performance in clinics and research and is usually achieved by ultrasound techniques. The main advantage of the ultrasound approach refers to the large number of cardiac features that it may quantify; however, the instrumentation is expensive and the measures should be taken by trained operators often in a clinical environment. All these aspects preclude the possibility to use this methodology for the monitoring of cardiac mechanics out of the laboratory setting and in daily life conditions. In those scenarios, a possible solution might be represented by the use of the seismocardiogram (SCG). This signal is the measure of the minute precordial vibrations produced by the beating heart and blood ejection, and indexes of cardiac mechanics may be derived from its analysis^1^.

SCG is easily detected by placing an accelerometer on the chest, and the measure may be taken also in a daily life environment^2,3^. The heart vibrations generate accelerations in the order of few milli-gs (1 g corresponds to the terrestrial gravitational acceleration, i.e. 9.81 ms^−2^) while the usual body movements result in accelerations 10 or 100 times higher (in the order of 0.1–1 g or more) that may mask the SCG signal when they occur. Thus, during spontaneous behavior SCG may be assessed whenever the subject’s physical activity is reduced (e.g. while sitting at the office desk, driving, standing at the bus stop, during sleep). In a previous study we recorded SCG for 24 hours in 5 healthy volunteers during a standard working day and we observed that the subjects remained spontaneously still, for at least 5 seconds, several tens of times per each hour of the day, thus allowing a frequent SCG assessment during the daily activities^2^. As expected, the situation was even better at night during sleep, when the SCG assessment was possible on a beat-to-beat basis for most of the nighttime.

From the methodological point of view, it should be considered that if the SCG recording is quite simple, the SCG analysis deserves particular attention. Indeed, because of its small amplitude it may be easily affected by artifact and, in addition, SCG morphology may change over time as a function of the respiratory phase and/or posture^1^. Thus the identification of fiducial points (FPs) in the waveform, needed for the estimation of the indices of cardiac mechanics, might be difficult.

If the number of SCG waveforms to be analyzed is limited (few minutes), a visual inspection of the signal may be sufficient to identify artifacts and fiducial points. However, this approach is not practicable with long term recordings, when tens of thousands beats should be scrutinized. In those cases an automatic procedure is necessary for the signal analysis, as we recently experienced.

Indeed, in recent years we had the opportunity to collect SCG data during sleep on ground and aboard the International Space Station, ISS, in the frame of the *Wearable Monitoring* project. More than 260,000 heart beat were recorded and so the automatic analysis was mandatory. It should be also considered that sleep recordings are not only characterized by a prolonged time duration, but also by a consistent and frequent change in the heart rate with bursts of bradycardia and tachycardia occurring in the different sleep stages which may easily span from 40 to 120 beats per minute.

So far, a number of algorithms have been proposed for the analysis of SCG. They were based on different approaches, including pattern or syntactic analysis^4–7^, wavelet^8^, and even neural network kernels^9^. However, they were designed and tested for the analysis of relatively short SCG recordings, collected in controlled conditions and with a relatively stable heart rate. Thus, although we cannot exclude that with modifications some of the above algorithms might have met our requirements, we decided to develop an ad-hoc procedure.

In this article, after preliminary descriptions of the space experiment from which data are derived and of the SCG morphology, we illustrate the details of the developed algorithm with a quantification of its performance.

## The “Wearable Monitoring” Project

In the period 2014–2015 we had carried out experiments on Earth and aboard the ISS, to gain insight into sleep mechanisms in microgravity as compared with on ground measures. The project, coordinated by Fondazione Don Gnocchi, was named “*Wearable Monitoring*” and was part of the Futura research mission of the Italian Space Agency, ASI^10^.

The project had two objectives. The first objective, of technological nature, consisted in the development of a new smart garment for the collection of the electrocardiogram (ECG), respiration, skin temperature and SCG, and in its validation during sleep in space.

The second objective, of biological nature, consisted in the use of the above device to collect data on aspects of sleep physiology in microgravity, including the beat-to-beat behavior of cardiac mechanics.

The rationale behind the study of cardiac mechanics in space is that the absence of gravity causes a deconditioning of the cardiac function because of the centralization of fluids and the loss of heart muscle mass due to the cardiac unloading induced by the abolition of the blood weight. In turn, experiments in rats evidenced that in unloaded hearts the myocardial cells may reduce their contractility^11^. To date the effects of all these factors on the cardiac mechanical function of the astronauts during spaceflights are still largely unexplored^12^, and further investigations are advisable for a deeper insight into life in space, for the astronauts’ safety, and, in case, for the development of proper countermeasures.

Our experiments allowed obtaining for the first time a continuous and prolonged monitoring of the cardiac function in space, and their execution during sleep provided data free from the behavioral interferences of the waking hours.

### The device developed for the experiment

**Figure 1:**
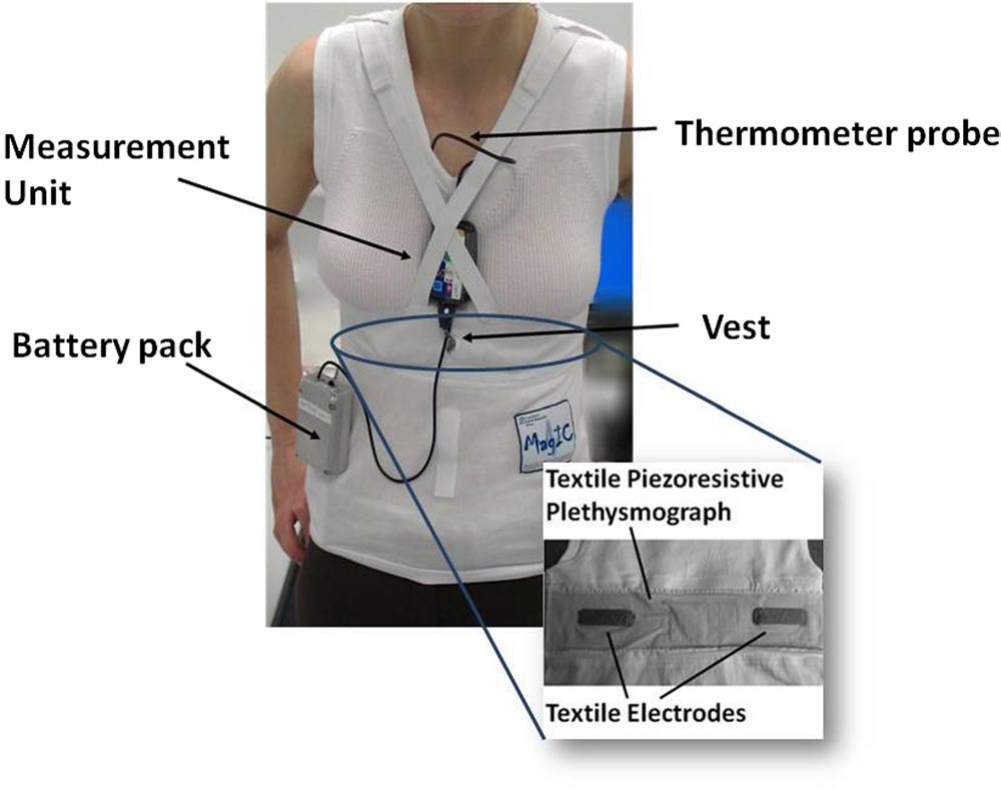
Model wearing the MagIC Space device and its components. Inset: detail of the textile sensors embedded in the inner part of the vest.

**Figure 2:**
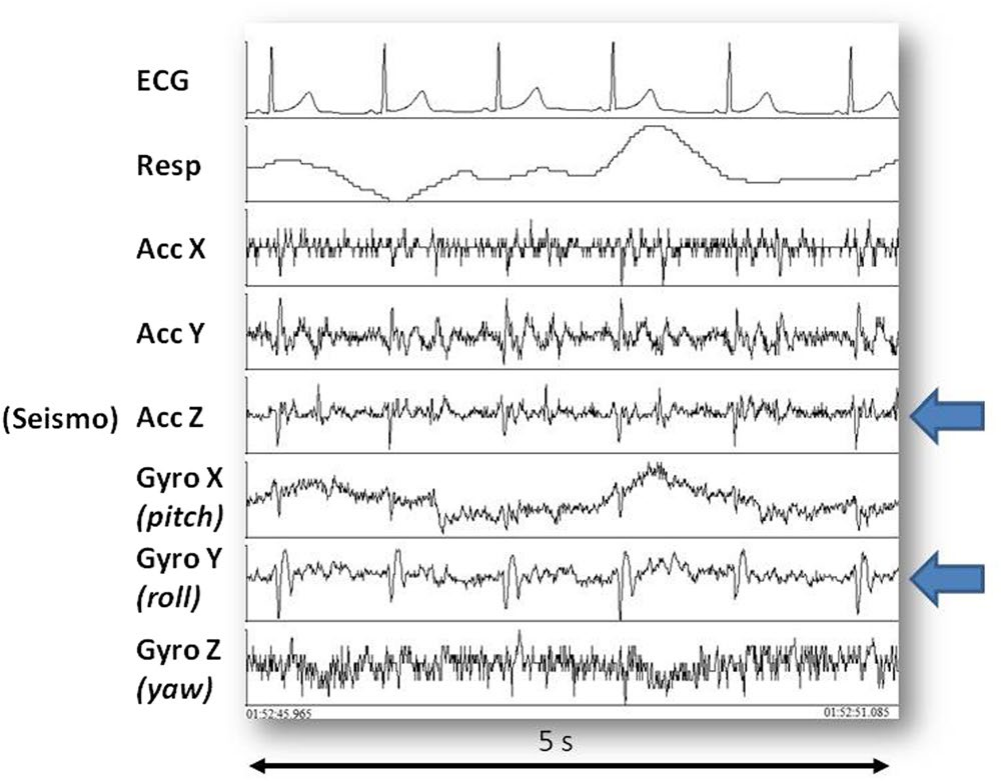
Example of recorded signals. From top to bottom: ECG, respiration, X,Y and Z accelerometric components, X, Y and Z gyroscopic components. Arrows indicate the accelerometer and gyroscope axes displaying synchronous patterns.

The device developed for the Wearable Monitoring project, MagIC-Space, was derived from a smart garment previously designed in our laboratory for the assessment of ECG, respiration and body motion (the MagIC system^13^). The original device was enhanced to include the additional measure of the SCG and skin temperature, to meet the space qualification requirements and to simplify the experiment setup and execution. MagIC-Space is illustrated in Fig. 1 and is composed of 1) a cotton vest embedding two textile sensors for the ECG measure and one textile plethysmograph for the respiratory assessment; 2) a miniaturized electronic module (the Measurement Unit) for the collection and storage of the signals from the vest sensors; this unit also contains a triaxial MEMS accelerometer (Freescale MMA8451Q, range: from +2 g to −2g, 14-bit) for the SCG detection and a triaxial gyroscope (STMicroelectronics L3GD20) for the measure of the body rotations; 3) a thermometric probe integrated into the vest for the assessment of the thorax skin temperature; and 4) a battery pack, containing two AA 1.5 V Lithium-ion batteries. All signals were sampled at 200 Hz after the anti-aliasing filtering. An example of data collected by the system is shown in Fig. 2.

Since all sensors were integrated into the garment, the astronaut had only to put on the system and start the monitoring, without taking care of the sensor positioning and wiring. This strategy led to a marked reduction in the setup time and complexity during the experiment preparation aboard the ISS.

### The experiment

The Italian crewmember (female, age: 38 years) participating in the ISS Expedition 42–43 was assigned the experiment. Seven inflight sleep recordings were made in the period January-June 2015 at mission days 53, 55, 56, 80, 116, 142 and 192. The experimental protocol was approved by the Ethical Review Board of our Institution and the NASA Institutional Review Board, received the signed informed consent of the astronaut, and all experiments were performed in accordance with relevant guidelines and regulations.

In each experimental session the astronaut (a) donned the MagIC-Space system and activated the data recording few minutes before going to sleep; (b) free floated for 5 minutes to obtain baseline pre-sleep values; (c) went to sleep; and (d) on the following morning, she stopped the recording at the wake up and transferred data from the Measurement Unit to a laptop aboard the ISS for the data downlink to Earth.

Two preflight and two postflight reference sleep recordings were also performed on the same subject in Cologne on October 2014 and July 2015.

Details on the collected data are shown in Table 1. It may be noted that the average sleep length was shorter in space than on Earth. This result is in line with previous observations available in literature^14^.

**Table 1:** Details of the collected data.

	In-Flight	On-Ground
No. sleep recordings	7	4
Total recording time	42 h 21 min	27 h 34 min
Average sleep length	6 h 3 min	6 h 53 min
Total no. recorded beats	166,400	97,400

## The Seismocardiogram

As mentioned, the SCG is the measure of the precordial vibrations produced at every heartbeat by the cardiac contraction and relaxation and by the blood ejection from the ventricles into the vascular tree.

**Figure 3:**
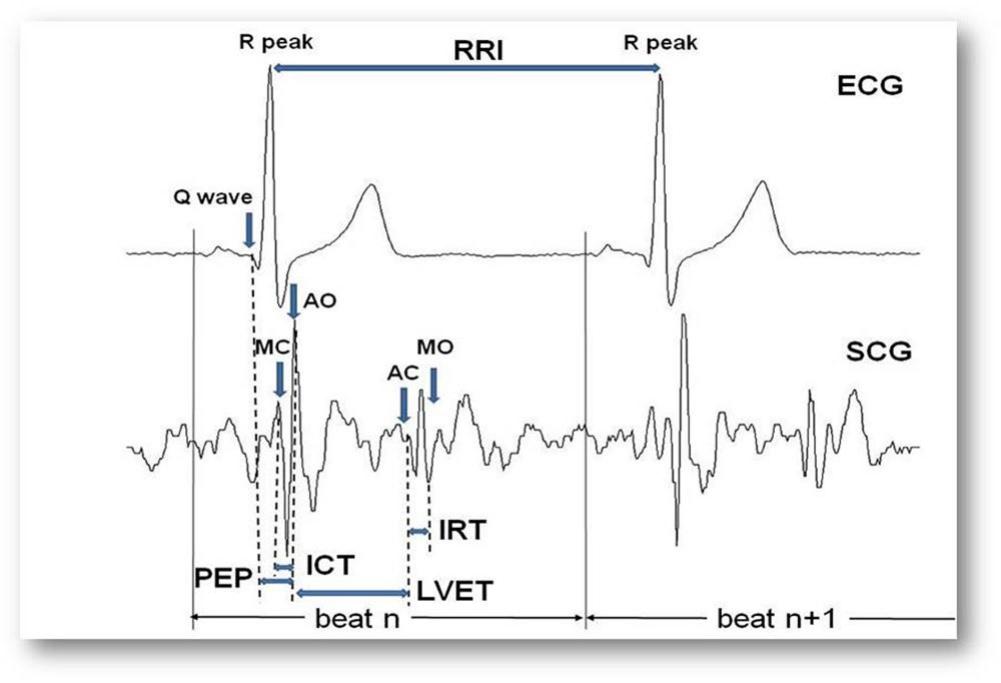
Typical SCG waveform with indication of the AO, AC, MO and MC fiducial points, and of the timings used for the estimation of the Cardiac Time Intervals. See text for the explanation of acronyms.

The typical SCG waveform, illustrated in Fig. 3, is characterized by a number of peaks and troughs. In particular, some of these displacements are associated with the opening and closure of the aortic valve, AO and AC, and the opening and closure of the mitral valve, MO and MC. The correspondence between those SCG FPs and the cardiac events was previously verified by simultaneous measurements with ultrasound techniques^15^.

Although SCG is a three-dimensional signal^1^, only the dorso-ventral component of acceleration (corresponding to the z-axes in our accelerometer, see Fig. 2) is prevalently considered for its assessment. In our analysis we comply with this traditional approach.

In the MagIC-Space device, the accelerometer was contained in the Measurement Unit and when the garment was worn this unit was kept in contact with the subject’s sternum by elastic straps (see Fig. 1). This arrangement guaranteed the SCG measure.

### Feature extraction: indexes of cardiac mechanics

As illustrated in Fig. 3, from the R and Q peaks in the ECG and the AO, AC, MO and MC fiducial points in the SCG waveform we may estimate the RR interval, RRI (i.e. the time interval between consecutive R peaks) and the Cardiac Time Intervals, CTIs, considered as indexes of cardiac mechanical function and defined as follows:

- Pre Ejection Period (PEP) = the time delay from the Q wave in the ECG to the AO fiducial point in the SCG;
- Isovolumic Contraction Time (ICT) = the time delay from MC to AO;
- Left Ventricular Ejection Time (LVET) = the time delay from AO to AC;
- Isovolumic Relaxation Time (IRT) = the time delay from AC to MO.

From the above parameters additional indexes may be derived, such as the PEP/LVET ratio, the TEI index, also termed Myocardial Performance Index (MPI), defined as (ICT + IRT)/LVET, and the LVET/RRI ratio.

PEP, ICT, LVET and the PEP/LVET ratio are indexes of heart contractility; IRT is an index of heart relaxation^16^;TEI index quantifies myocardial performance^17^, LVET/RRI quantifies the proportion between systolic and diastolic phases of the cardiac cycle, being this proportion another index of cardiac function.

## The Algorithm For The SCG Analysis

The algorithm may be subdivided into three phases:

1. Data segmentation and artifact removal
2. SCG fiducial point extraction
3. Congruency check & CTI Estimation

Obviously, we may obtain correct CTI values only from a good performance of the algorithm in detecting the AO, AC, MO and MC fiducial points. Given the large number of available heart beats, the algorithm was designed to maximize its precision, i.e. its ability to correctly identify true FPs, even at the cost of false negatives.

**Figure 4:**
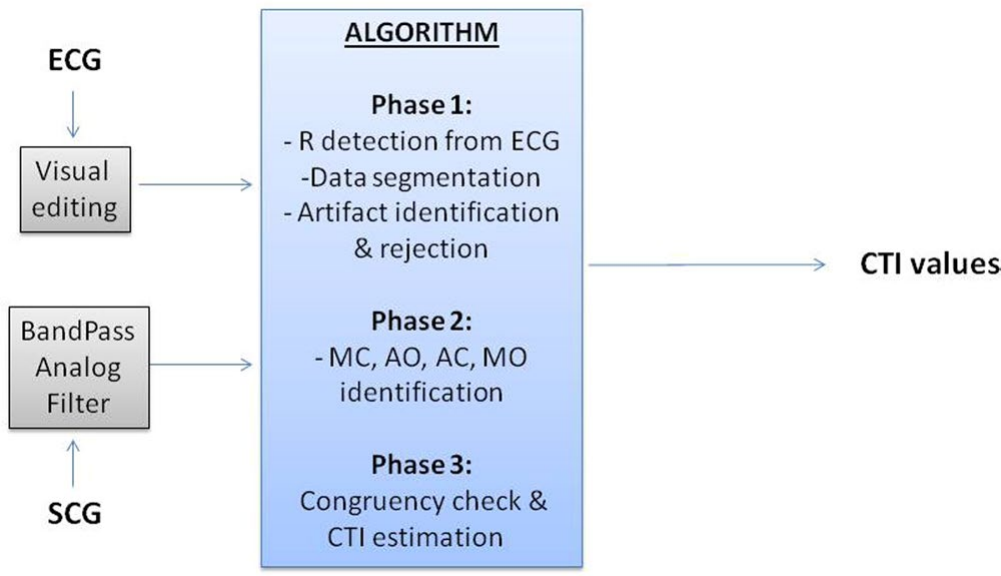
Scheme of the whole signal processing and of the algorithm phases.

The algorithm makes use of both ECG and SCG signals. As schematized in Fig. 4, for each recording ECG and SCG were pre-processed before feeding the algorithm. In particular, the ECG trace was manually edited from artifacts by a proprietary software previously developed (the average ECG artifact rate was <0.5%), while the SCG signal was filtered by a forward-reverse application of a band pass Butterworth filter (5–40 Hz) to remove baseline drifts and noise.

In the following description, the subscript “_*Rdelay*_” indicates the time delay of a given FP or event with respect to the ECG R peak, e.g. AO_*Rdelay*_ means the time delay of AO from the corresponding R peak. All abbreviations used in the algorithm description are summarized in Table 2.

**Table 2:** List of abbreviations used in the algorithm description. The superscript ^i^ indicates the current beat, ^i+1^ the first next beat and ^i+2^ the second next beat.

**Figure 5:**
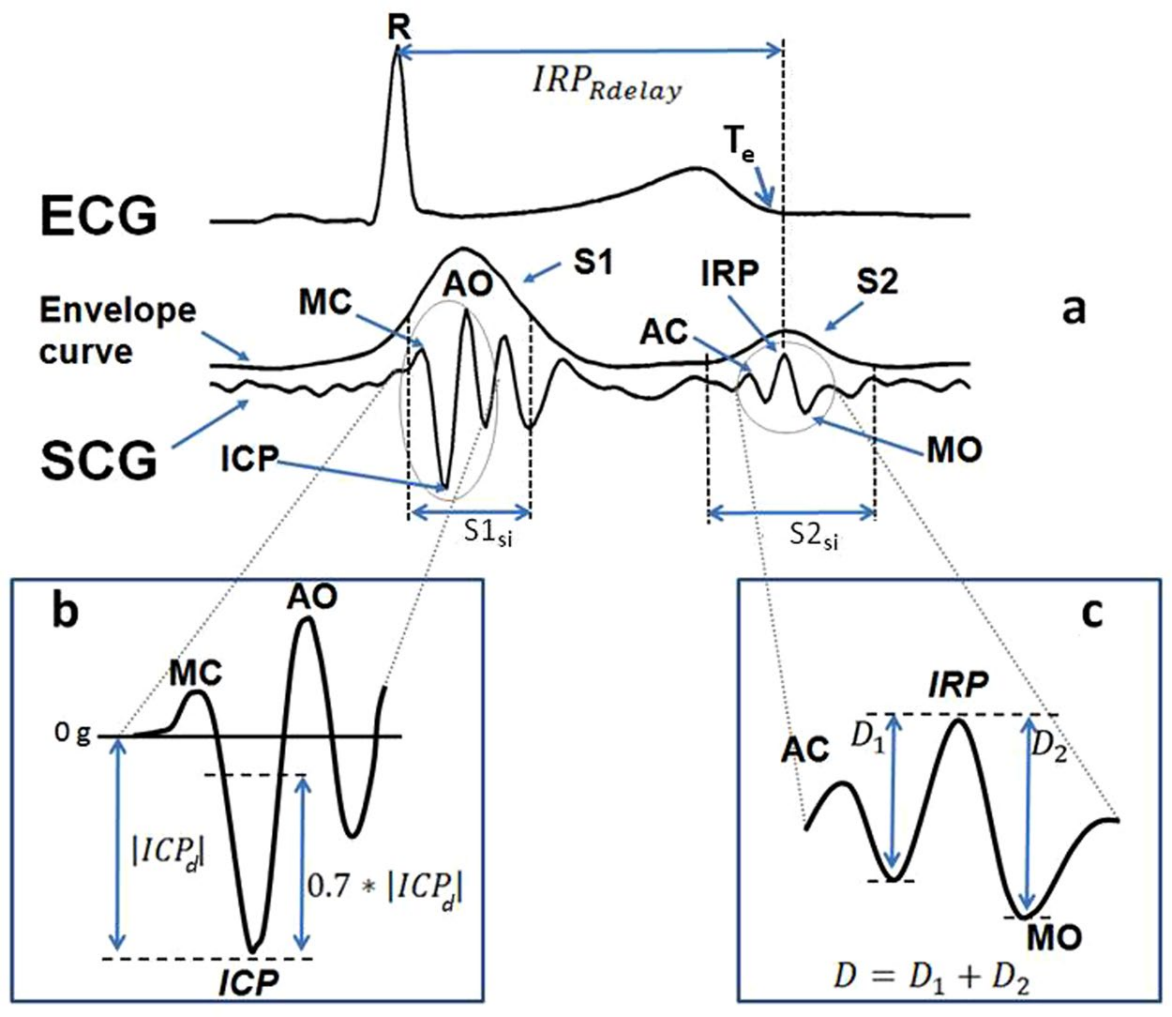
Scheme of FP identification. Panel a: ECG with the indication of the Te point, SCG with its envelope curve and indication of the S1 and S2 areas, MC, AO, AC and MO fiducial points, ICP and IRP anchor points and definition of the IRP_Rdelay_ used for the AC and MO identification. Inset b: definition of the threshold points used for the estimation of MC and AO in the S1_si_ window. Inset c: definition of the D1 and D2 distances used for the scoring of the peaks in the S2_si_ window for the identification of the IRP anchor point used for the localization of AC and MO.

AC = Closure of the aortic valve (fiducial point)
AC_*Rdelay*_ = *Time position of AC with respect to the R peak (in ms)*
AFP_*Rdelay*_ = Average FP_*Rdelay*_ estimated in the 5 beats preceding or following the current beat
AO = Opening of the aortic valve (fiducial point)
MC = Closure of the mitral valve (fiducial point)
MO = Opening of the mitral valve (fiducial point)
*CFTP* = *Coarse FP Time Position: FP timing with a 5* *ms precision (in ms)*
D = D1 + D2
D1 = Distance (in amplitude) between IRP and the adjacent left minimum (in mg), see Fig. 5 panel c
D2 = Distance (in amplitude) between IRP and the adjacent right minimum (in mg), see Fig. 5 panel c
FP = Fiducial Point (either AC, AO, MC or MO)
FP_*Rdelay*_ = *Time position of the generic FP with respect to the R peak (in ms)*
*HRFTP* = *HiRes FP Time Position: FP timing with 1* *ms precision (in ms)*
ICP = Isovolumic Contraction Point (anchor point used for detection of MC and AO)
|ICP_d_| = Absolute value of the ICP distance from the 0 g baseline (in mg)
*ICP*_*Rdelay*_ = *Time position of ICP with respect to the R peak (in ms)*
*ICP*_*Rdelay*_^*ref*^ = *ICP*_*Rdelay*_ *observed in the closest valid beat (the reference)*.
IRP = Isovolumic Relaxation Point (anchor point in S2 used for detection of AC and MO)
*IRP*_*Rdelay*_ = *Time position of IRP with respect to the R peak (in ms)*
*IRP*_*Rdelay*_^*ref*^ = *IRP*_*Rdelay*_ *observed in the closest valid beat among the last 20 beats (the reference)*.
*RRI* = *RR Interval (in ms)*
S1 = Peak in the SCG envelope corresponding to the first heart sound
S1_si_ = 50 ms SCG segment starting after 25 ms from the R peak where ICP, MC and AO are searched for
S2 = Peak in the SCG envelope corresponding to the second heart sound
S2_si_ = 60 ms SCG segment centered on Te where IRP, AC and MO are searched for
Te = End of T wave
*Te*_*Rdelay*_ = *Time position of Te with respect to the R peak (in ms)*

### Phase 1–Data segmentation and artifact removal

Goal of this phase was to split each recording into individual heart beats and identify and remove both gross artifacts caused by marked body movements and smaller artifacts due to smooth bumps, small movements, shifts between clothes layers, etc.

In detail

1. In each valid ECG complex the position of the R peak was identified and stored.
2. On the basis of the R peak timing, the ECG and the SCG were then split into individual heart beats. We considered as a heart beat a signal segment starting from −200 ms from the relevant R peak till −200 ms from the following R peak (see Fig. 3). The −200 ms offset was adopted to include in the beat also the corresponding ECG P wave, although this information was not considered in the present study.
3. Gross artifacts were identified by checking the SCG amplitude and variance within each heart beat. Beats with the maximal peak to peak amplitude greater than 50 mg or a variance greater than 28 mg^2^ were excluded from the subsequent analyses. Those threshold values were empirically derived from the analysis of a sample of beats randomly extracted from all recordings.
4. Smaller artifacts were identified in the remaining beats by computing the envelope of the SCG. The envelope was obtained by calculating the absolute value of the SCG signal and then filtering the output by a 31-sample FIR filter with a triangular window. As shown in Fig. 5(a) the typical envelope curve is characterized by two main peaks, S1 and S2, corresponding to the first and second heart sound.

We considered as normal the beats in which the envelope amplitude of S1 was higher than that of S2 **AND** + 10 ms ≤S1_Rdelay_ ≤ +160 ms **AND** +300 ms ≤ S2_Rdelay_ ≤ +480 ms.

Beats not satisfying all the above three criteria were considered as artifacts and discarded from the subsequent analyses.

### Phase 2- SCG Feature extraction

Goal of this phase was to identify in each beat the FPs associated with the opening and closure of the aortic and mitral valves. The search for each FP was done in two steps. In the first step we localized the interval where the FP should have been searched for. This was done by identifying two anchor points in the SCG waveform, the Isovolumic Contraction Point, ICP, to estimate MC and AO, and the Isovolumic Relaxation Point, IRP, to estimate AC and MO (see Fig. 5). In the second step, the FP was finally localized by a pattern analysis starting from the relevant anchor point. This policy was adopted since we observed that the selected anchor points are the clearer displacements within the SCG waveform. As their names suggest, they correspond to the SCG vibrations produced during the isovolumic contraction and the isovolumic relaxation phases of the heart cycle, respectively. Concerning the ICP and IRP localization it should be pointed out that while the relative position of ICP with respect to the R peak is mostly independent from the heart rate, this is not the case for the IRP position which, on the contrary, is largely influenced by changes in the beat length. This aspect was taken into account in the design of the algorithm.

### MC And AO Identification

In the SCG signal under the S1 envelope peak we localized a search interval, S1_si_, starting and ending 25 and 75 ms after the R peak respectively. The ICP was the deepest minimum within S1_si_ that satisfies the following condition: |ICP_*Rdelay*_ − ICP_*Rdelay*_^ref^ |≤ +30 ms, being ICP_*Rdelay*_^ref^ the ICP_*Rdelay*_ observed in the last valid beat before the current beat and taken as a “reference”. That is, the ICP was accepted if it occurred within a given delay from the R peak **and** if this delay was sufficiently close to that observed in the previous reference beat.

In the beats were the ICP was identified, the AO fiducial point was assumed to be the first peak following the ICP within 50 ms and with a distance (in amplitude) from ICP greater or equal to 0.7*|ICP_*d*_|, being ICP_*d*_ the ICP peak amplitude (see Fig. 5(b)). This threshold was set in order to exclude spurious peaks occasionally occurring in proximity of the anchor point. The MC fiducial point was assumed to be the first peak preceding the ICP within 50 ms and satisfying the same amplitude rule of AO.

### AC And MO Identification

For the localization of the IRP anchor point and then of the AC and MO FPs, we identified, under the S2 envelope peak, a SCG search interval S2_si_ defined as the 60 ms SCG segment centered on the end of the ECG T wave (Te). Te was determined through the procedure proposed in^18^. This strategy simplified the search for IRP even in presence of important changes in the cardiac rhythm. Indeed, while Te_Rdelay_ may change as a function of the cardiac interval, in healthy subjects IRP remains quite close to Te.

IRP was the highest peak in S2_si_ which met two criteria. The first criterium verifies that the IRP peak be sufficiently pronounced, the second criterium verifies that IRP_Rdelay_ be congruent with the IRP_Rdelay_ in the adjacent beats.

In detail:

#### First criterium

Being D the sum of the two distances, D1 and D2 between the IRP peak and the adjacent left and right minima (see Fig. 5(c)), we verified that D ≥ 7 mg.

#### Second criterium

In case at least one of the 20 beats preceding the current beat contained a valid IRP, the IRP_Rdelay_ of the valid beat closest to the current beat was taken as reference, IRP_*Rdelay*_^*ref*^, and we verified that

$$|{{\rm{IRP}}}_{Rdelay}-{{{\rm{IRP}}}_{Rdelay}}^{ref}|\le 20\,{\rm{ms}}$$

In case no reference beat was available among the previous 20, e.g. because of a sequence of artifacts, we checked the congruency of the current beat vs. the subsequent two beats, provide the IRP could be estimated in those beats and their RR intervals was “relatively” stable, i.e.

$$|{{\rm{RRI}}}^{i}-{{\rm{RRI}}}^{i+1}|\le 100\,{\rm{ms}}\,{\boldsymbol{AND}}\,|{{{\rm{IRP}}}_{Rdelay}}^{i}-{{{\rm{IRP}}}_{Rdelay}}^{i+1}|\le 20\,{\rm{ms}}\,{\boldsymbol{AND}}$$

$$|{{\rm{RRI}}}^{i+1}-{{\rm{RRI}}}^{i+2}|\le 100\,{\rm{ms}}\,{\boldsymbol{AND}}\,|{{{\rm{IRP}}}_{Rdelay}}^{i+1}-{{{\rm{IRP}}}_{Rdelay}}^{i+2}|\le 20\,{\rm{ms}}$$

If the IRP anchor point was identified, because both the above two criteria were met, AC was the first peak preceding IRP if

$$10\,{\rm{ms}}\le |{{\rm{IRP}}}_{{Rdelay}}-{{\rm{AC}}}_{{\rm{Rdelay}}}|\le 40\,{\rm{ms}}$$

and MO was the next trough following IRP if

$$10\,{\rm{ms}}\le |{{\rm{MO}}}_{{\rm{Rdelay}}}-{{\rm{IRP}}}_{{Rdelay}}|\le 30\,{\rm{ms}}$$

**Figure 6:**
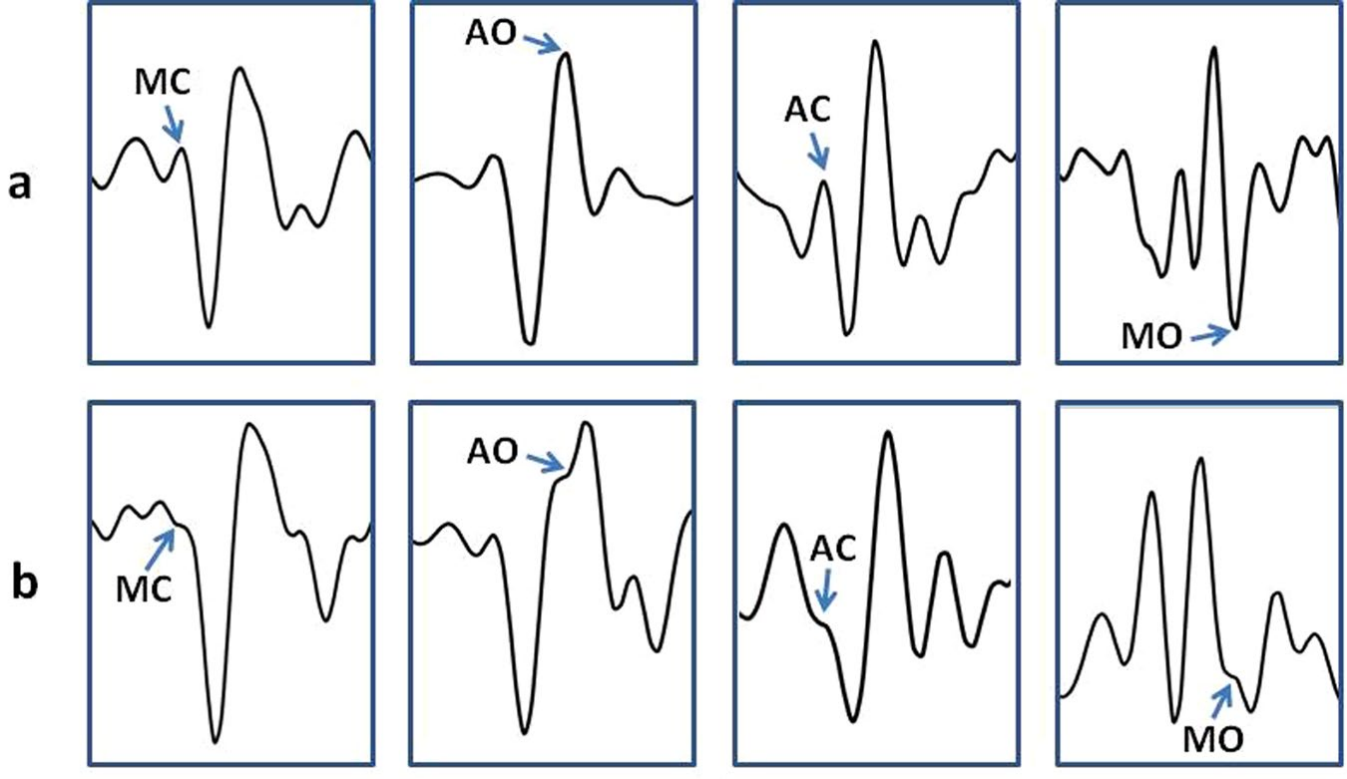
Panel a: Examples of beats in which the MC, AO, AC and MC fiducial points are characterized by the expected clear-cut peaks (for MC, AO and AC) or trough (for MO). Panel b: other beats in which the FPs are marked only by an inflection point.

**Figure 7:**
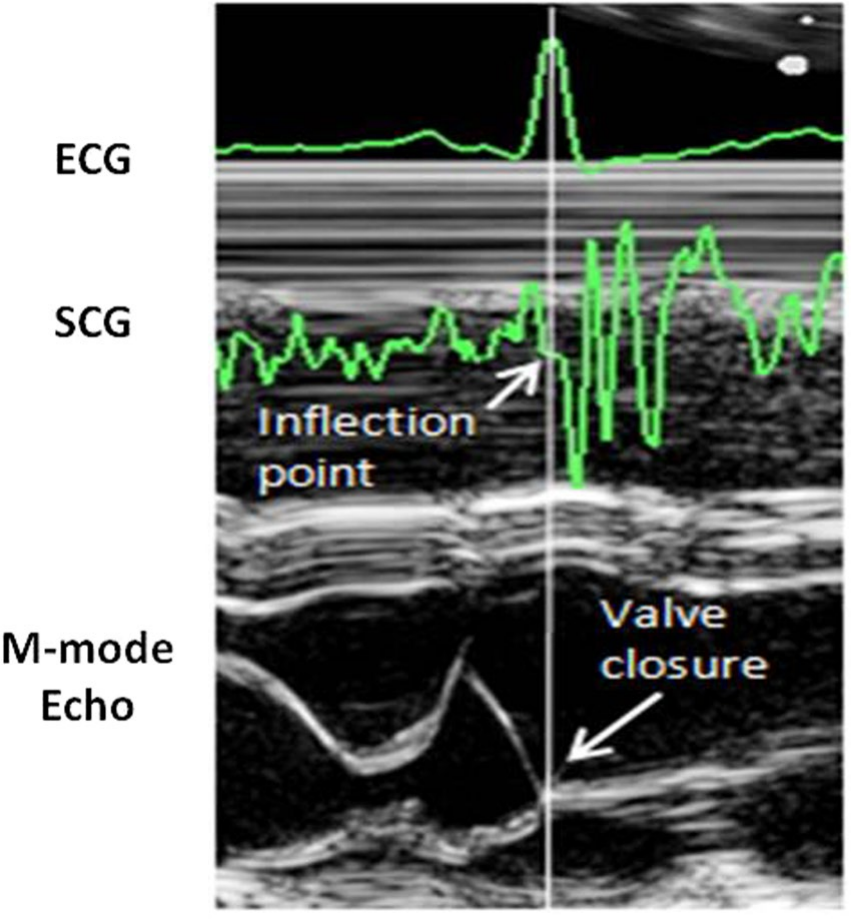
Example of SCG inflection point corresponding to the mitral valve closure. M-mode ultrasoung image from a healthy subject.

Sometimes a variant to the FP identifications described above was applied. As shown in Fig. 6, AO, AC and MC do not always appear as clear peaks and MO as a clear valley. In about 6% of the analyzed beats, we observed an inflection point in the area where the target peak or valley was expected (on the basis of the FP position detected in the previous beats) and in those cases the inflection point was taken as marker of the FP. In the past we already observed this phenomenon in different subjects during experimental laboratory recordings. One example of the correspondence between MC inflection point and the real closure of the mitral valve is shown in Fig. 7. Data refers to a simultaneous SCG and M-mode ultrasound measure in a healthy volunteer. Although the origin of this phenomenon is still unexplored, we speculate that it might depend on a transient slight physiological de-synchronization between left and right heart mechanics.

### Improvement of FPs Time Resolution

**Figure 8:**
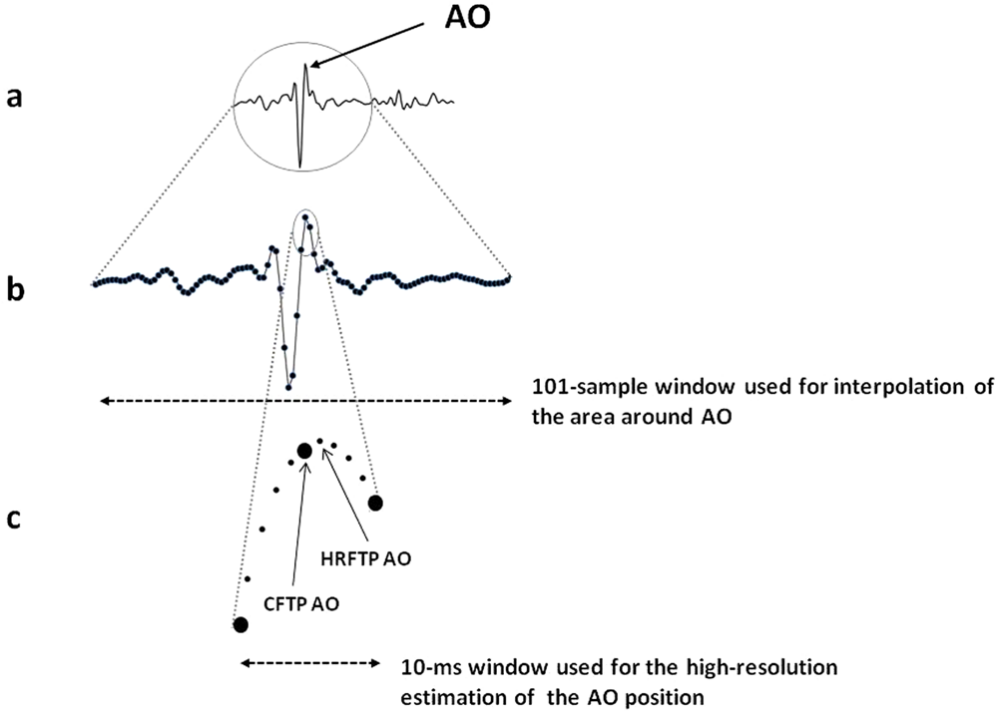
Schematization of the signal interpolation and re-sampling for the 1-ms resolution assessment of FPs. In this representation the FP considered is AO. Panel a: the entire SCG waveform. Panel b: detail of the data window used for the interpolation. Panel c: the small data window used for the hi-res localization of AO, the larger circles represent the original low resolution samples and the smaller circles the final high resolution samples, CFTP = Coarse FP Time Position, HRFTP = Hi-Res FP Time Position.

At this stage of the procedure, the time position of each detected FP was identified with a 5 ms resolution (the original signal was sampled at 200 Hz). Let’s term this time “Coarse FP Time Position”, CFTP. We further refined the FP estimate by obtaining a “HiRes FP Time Position”, HRFTP, with the resolution of 1 ms. The Nyquist–Shannon sampling theorem guarantees that this was possible because the signal is bandlimited to 40 Hz and sampled at 200 Hz, thus the interpolation can accurately reconstruct the signal and determine the actual locations of the FPs at a higher resolution. As schematized in Fig. 8, for each FP we considered a 101-sample window centered on its CFTP, then we interpolated the data window by the sync function, re-sampled the interpolating function at 1 kHz and repeated the FP estimation on the hi-res samples in a 10 ms window centered on the CFTP.

A clarification: although only the 10 ms windows was used to refine the FP time location, the sync function was applied on the larger 101 sample window to minimize possible edge effects on the interpolated curve in the target interval.

The hi-res time of occurrence of valid AO, AC, MO and MC fiducial points were stored for the subsequent analysis.

### Phase 3–Congruency check and CTI estimation

In order to detect and eliminate possible residual outliers in the identified FPs, two additional congruency checks were made. The first directly on the FPs and the second on the derived CTIs.

As to the first check, each FP_Rdelay_ was compared with the average FP_Rdelay_, AFP_Rdelay_ computed over the preceding 5 beats. In absence of at least one preceding valid value, the current FP_Rdelay_ was compared with the AFP_Rdelay_ computed over the subsequent 5 beats. The FP was accepted if the difference between the FP_Rdelay_ of the current beat and AFP_Rdelay_ was lower or equal to 10 ms for MC and AO and 20 ms for AC and MO.

Then, from the beat-to-beat series of RRI, AO, AC, MO and MC, the CTIs were computed as indicated in section 3.

Finally, the second congruency check was done on the derived CTIs. In this case we verified that the difference between each CTI (PEP, ICT, LVET and IRT) and the average value of the same CTI observed in the preceding, or following 5 beats, analogously to the first check, was lower or equal to 10 ms for PEP and 20 ms for the remaining indexes. In case a CTI exceeded this limit, the FPs which concurred to its estimation were descarted (e.g. in case a given LVET exceeded the thresholds, the same LVET and the corresponding AO and AC fiducial points used for its estimation were discarded from the final data series).

## Statistical evaluation of algorithm performance

As mentioned, the main aspect of the procedure to be verified was the algorithm ability to correctly identify true FPs, namely, its precision. This quantity is statistically estimated by the Positive Predictive Value, PPV, defined as TPos/(TPos + FPos), where TPos are True Positives and FPos are False Positives^19^. PPV may range from 0 to 1 (or 0%-100% when expressed in percent) and was estimated as follows.

- One inflight and one on-ground recording were randomly selected from the whole dataset and analyzed by the algorithm.
- In each recording, for any given FP.

- A sample of beats in which the FP was identified by the algorithm was randomly selected; the sample size was of 2783 beats for the inflight data and 2346 beats for the on-ground data, corresponding to 10% of the valid beats in the respective recording. On the basis of the simulation on a million trials described in the Supplementary Material online, it resulted that those sample sizes lead to a possible max error in the PPV estimation of 1.1%.
- The TRUE or FALSE correctness in the FP localization was checked by an operator who compared the SCG waveform with the traditional template proposed by Crow and colleagues^15^.
- From the TRUE Positives and the FALSE Positives the PPV index was computed.

As shown in Table 3, PPV is extremely high (>99%) in both the recordings. The minimum was observed for the MO identification on on-ground data (99.2%), indicating, however, a misclassification in only 0.8% of the samples.

**Table 3:** PPV values, in percent, for each fiducial point analyzed by the algorithm, separately reported for the inflight and on-ground recording considered for the test.

	In-Flight	On-Ground
Events	PPV	PPV
MC	99.9	99.9
AO	100	99.9
AC	99.9	99.4
MO	99.6	99.2

These positive figures confirm the ability of the algorithm to identify the FPs and make trustable the value of their incidence shown in the next section.

## Signal quality and fiducial points incidence

The signal quality (defined as 100 minus the artifacts rate in percent) and the rate of the four FPs derived from the analysis of the two recordings are shown in Table 4.

**Table 4:** Signal quality and rate of estimation of the SCG fiducial points in the the inflight and on-ground recording considered for the test. The signal quality is expressed as percent of artifact-free beats with respect to the total number of available beats. The rate of estimation of the fiducial points is expressed in percent of the artifact-free beats.

	In-Flight	On-Ground
SCG quality (artifact-free beats)	98.2	91.0
***SCG fiducial points***		
MC	94.3	99.0
AO	99.7	96.7
AC	92.5	77.7
MO	96.6	72.9
All 4 fiducial points	85.1 (23,258 beats)	64.2 (13,716 beats)

From the table it appears that the SCG quality remained quite high in space (98.2%) with a reduction to 91% on-ground. This reduced quality on Earth was mainly due to artifacts caused by the changes in the body position occurring during sleep because of the contact with the bed. Conversely, in space the astronaut sleeps inside a sleeping bag that is only loosely tied to the ISS structure, thus the body fluctuates with a consequent drastic reduction in the artifacts rate.

Table 4 also reports the estimation rate for each of the four SCG fiducial points. It is apparent that each FP could be estimated in more than 92% of the beats occurring in the in-flight recordings. The rate is lower for the closure of the aortic valve and the opening of the mitral valve recorded on Earth (77.6% and 72.9%, respectively). This finding may be explained by considering the negative effects of possible residual sub-threshold artifacts still caused by the changes in the body position on ground, which may corrupt the SCG profile and limit the FP identification.

The rate of beats in which all the four FP were estimated was again higher in space than on ground (85% vs. 64%). However, it should be noted that these rates indicate that all the four FPs were present in more than 23,000 beats in space, and more than 13,000 beats on ground, a quantity more than sufficient for a detailed estimation of cardiac mechanics during sleep in microgravity and at 1 g.

**Figure 9:**
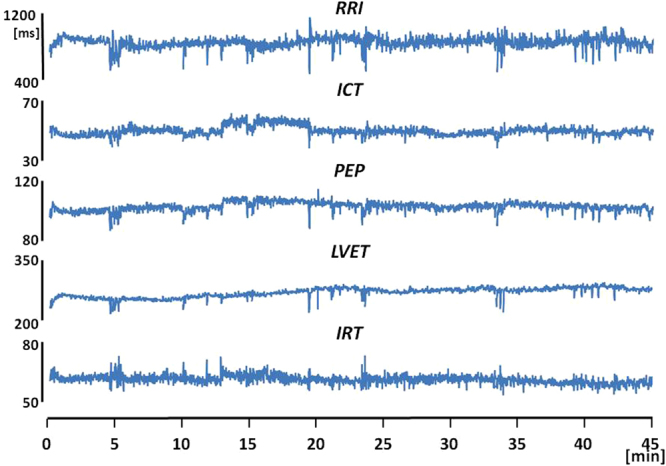
A 45-min segment of beat-to-beat RRI and indexes of cardiac mechanics excerpted from the first sleep recording made aboard the ISS.

As an example of the algorithm output, a 45-min segment of beat-to-beat RRI and CTIs derived from the first in-flight sleep recording is shown in Fig. 9. This is the first representation of the beat-to-beat variability of indexes of cardiac mechanics during sleep either on ground and in microgravity. The analysis of the CTIs dynamics estimated from the Wearable Monitoring data is currently in progress. The description of results and their interpretation will be subject of a separate article.

## Discussion

We recently had the opportunity to collect a large amount of SCG and ECG data during sleep in space and on Earth as part of an experimental campaign organized by the Italian Space Agency.

SCG has never been investigated during sleep neither on Earth. Handling SCG sleep recordings implies the analysis of a large number of beats and the frequent and marked variations of cardiac rhythm that characterize the different sleep stages. During sleep physical activity is minimized and thus SCG and the derived indexes of cardiac mechanics may be estimated on a beat-to-beat basis for most of the sleep duration. This means that sleep is also a unique opportunity to investigate short and long term features of cardiac mechanics variability

No algorithm is currently available for the processing of the SCG sleep recordings, thus our software represents the first procedure specifically designed for this scenario. The algorithm we developed was composed of three parts. In the first, artifacts were identified and removed on the basis of the signal amplitude and variance, and of the envelope morphology; in the second part the FPs were identified on the basis of a pattern analysis; in the third part, the indexes of cardiac mechanics were derived from the FPs. From the methodological perspective, the algorithm includes elements of novelty never adopted in the past: 1) the use of the T wave for the detection of the AC and MO fiducial points; 2) the interpolation-upsampling procedure, providing the FPs time localization with 1ms resolution from a signal sampled at 200 Hz (this approach allows a significant reduction in the memory allocation with respect to a 1 kHz sampling of the entire signal); 3) the inclusion of the inflection points as possible markers of FPs.

The evaluation of the algorithm precision led to an extremely good score with PPV values ≥99.2%. This positive result also depends on the fact that the algorithm was designed to maximize the true positives, as indicated in section 4.

The application of the algorithm to the experimental data provided us with the automatic identification of all four FPs in more than 23,000 beats in the space recording and 13,000 beats in the on-ground recording, a task impossible to have been achieved by manual procedures.

It should be mentioned that the algorithm includes several threshold tests and most of the threshold values were set empirically, on the basis of our past experience in working with the SCG signal. We believe that in a future refinement of the algorithm a finer tuning of these thresholds is possible, with a further enhancement of the algorithm performance. Another possible future enhancement of the algorithm may derive from the use of the gyroscope. In the MagIC-Space device we included a gyroscope aside the accelerometer to detect major body rotations. When we looked at the collected data, however, we realized that the gyroscope was able to detect also small body rotations associated to the heart activity. This aspect is illustrated in Fig. 2 where simultaneous SCG and gyroscope data (indicated by the arrows) are shown. It is apparent a synchronization between the systolic phase of the SCG and a specific pattern in the y axis (roll) of the gyroscope indicating a micro-rotation around the longitudinal (head-foot) axis in phase with the heart contraction. A first investigation of the relationship between SCG and gyroscope signals recently appeared in^20^. Yet, from the methodological perspective, the above pattern in the gyroscope data might also be exploited as an additional anchor point for the identification of the MC and AO points in the SCG tracings.

Finally, a note on the algorithm portability. Although our procedure was focused on the astronaut, it is conceivable that it might be used also for the analysis of data from other subjects, after the change of some parameters to adapt it to possible individual differences in the SCG morphology. In the opposite direction, it may be also hypothesized that the procedure was so tightly tailored on the SCG waveform of the astronaut to preclude its adaptation to any other subject with reasonable efforts. Thus, without the presumption to address this issue at large, we carried out an ancillary analysis aimed at checking on whether a tuned version of the algorithm might be obtained by the only change of threshold values and might be used to analyze data of subjects different from the astronaut. Details of the analysis are reported in the part II of the Supplementary Material on line. In short, two additional sleep recordings were taken from two healthy volunteers, and an effective tuning of the algorithm was obtained by the change of two threshold values of the algorithm in one subject and three values in the other subject. The adapted procedures yielded FPs identifications characterized by elevated precision, with PPV always >99%.

These findings clearly demonstrate that the algorithm may be tuned for the analysis of data from other subjects without requiring any structural change. The extent of the algorithm portability to a larger population and to selected subgroups of subjects (classified by gender, age, body mass index, health status, etc.) remains to be investigated in a future study.

## **C**onclusions

The possibility to investigate cardiac mechanics through the SCG analysis open interesting perspectives in terms of understanding of the cardiovascular function in daily life and in extreme environmental conditions such as in microgravity. Thus an appropriate analysis of the SCG signal is essential. The positive results we obtained by the performance evaluation of the new algorithm, provide evidence, for the first time, that an automatic detection of the four SCG fiducial points associated with the opening and closure of aortic and mitral valves (from which indexes of cardiac mechanics may be derived) is feasible in long lasting recordings, taken out of the laboratory setting, and even in presence of wide heart rate swings. In addition, the positive results obtained by the ancillary analysis provides a first positive support to the algorithm portability, although its bounds should be further evaluated.

### Data Availability

The astronaut datasets analysed during the current study is not publicly available according to the Data Privacy and Confidentiality rules of the NASA Institutional Review Board. The remaining datasets are available from the corresponding author on reasonable request.

## Electronic supplementary material

Supplementary Material

## References

[1] Inan O (2015). Ballistocardiography and Seismocardiography: A Review of Recent Advances. J. Biomed. Health Inform..

[2] Di Rienzo, M., Meriggi, P., Vaini, E., Castiglioni, P. & Rizzo, F. *24h Seismocardiogram Monitoring In Ambulant Subjects*. In: Proc Conf IEEE EMBS, San Diego, 2012, 5050-5053 (IEEE Press, 2012). 10.1109/EMBC.2012.6347128 23367063

[3] Di Rienzo M (2013). Wearable seismocardiography: towards a beat-to-beat assessment of cardiac mechanics in ambulant subjects. Auton. Neurosci..

[4] Giorgis L (2012). Optimal algorithm switching for the estimation of systole period from cardiac microacceleration signals (SonR). IEEE Trans. Biomed. Eng..

[5] Khosrow-Khavar, F., Tavakolian, K., Blaber, A. & Menon, C. Automatic and Robust Delineation of the Fiducial Points of the Seismocardiogram Signal for Non-invasive Estimation of Cardiac Time Intervals. *IEEE Trans. Biomed. Eng*. Oct 12, (2016) [Epub ahead of print]. 10.1109/TBME.2016.2616382 28113202

[6] Javaid, A. Q. *et al*. Quantifying and Reducing Motion Artifacts in Wearable Seismocardiogram Measurements during Walking to Assess Left Ventricular Health. *IEEE Trans. Biomed. Eng*. Aug 16. (2016) [Epub ahead of print]. 10.1109/TBME.2016.2600945 PMC5444999 27541330

[7] Shafiq, G., Tatinati, S., Ang, W. T. & Veluvolu, K. C. Automatic Identification of Systolic Time Intervals in Seismocardiogram. *Sci Rep*. *6*, 37524, 10.1038/srep37524 (2016). 10.1038/srep37524 PMC5118745 27874050

[8] Sandham W, Hamilton D, Fisher A, Xu W, Conway M (1998). Mulriresolution Wavelet Decomposition of the Seismocardiogram. IEEE Trans. Sig. Proc..

[9] Poliac, M. O., Zanetti, J., Salerno, D. & Wilcox, G. L. Seismocardiogram (SCG) interpretation using neural network. In *Proc. IEEE Symp on The Engineering of Computer-Based Medical Systems* 288–295 Computer Society Press (1998).

[10] Di Rienzo, M., Vaini, E. & Lombardi, P. Wearable Monitoring: a Project for the Unobtrusive Investigation of Sleep Physiology Aboard the International Space Station. In: Proc. Computing in Cardiology 2015; 42, 125–128 (2015).

[11] Ito K (2003). Contractile Reserve and Calcium Regulation Are Depressed in Myocytes From Chronically Unloaded Hearts. Circulation..

[12] Aubert AE (2016). Towards human exploration of space: the THESEUS review series on cardiovascular, respiratory, and renal research priorities. Nature PJ Microgravity.

[13] Di Rienzo, M. *et al*. MagIC System: a New Textile-Based Wearable Device for Biological Signal Monitoring. Applicability in Daily Life and Clinical Setting. In: Proc. IEEE EMB Conference 2005, Shanghai. 7167–7169, IEEE Press. (2005). 10.1109/IEMBS.2005.1616161 17281930

[14] Elliot AR (2001). Microgravity reduces sleep-disordered breathing in humans. Am. J. Resp. Crit. Care Med..

[15] Crow RS, Hannan P, Jacobs D, Hadquist L, Salerno DM (1994). Relationship between Seismocardiogram and Echocardiogram for Events in Cardiac Cycle. Am. J. Noninvasive Cardiology.

[16] Marcus FI (2007). Accelerometer-Derived Time Intervals during Various Pacing Modes in Patients with Biventricular Pacemakers: Comparison with Normals. PACE.

[17] Bruch C (2000). Tei-Index in patients with mild-to-moderate congestive heart failure. Eur Heart J.

[18] Laguna P (1990). New algorithm for QT interval analysis in 24-hour Holter ECG: performance and applications. Med. Biol. Eng. Comput..

[19] Pepe M. S. The statistical evaluation of medical tests for classification and prediction. Oxford University Press (2003).

[20] Jafari Tadi, M. *et al*. Gyrocardiography: A New Non-invasive Monitoring Method for the Assessment of Cardiac Mechanics and the Estimation of Hemodynamic Variables. *Sci Rep* **7**(1), 6823, doi:10.1038/s41598-017-07248-y (2017). 10.1038/s41598-017-07248-y PMC5533710 28754888

