# Supplementary material for: An algorithm for the beat-to-beat assessment of cardiac mechanics during sleep on Earth and in microgravity from the seismocardiogram
Source: Sci Rep. 2017 Nov 15;7:15634. doi: 10.1038/s41598-017-15829-0 (PMC5688070; doi:10.1038/s41598-017-15829-0)
Supplement: Supplementary file 1 — Supplementary Material [file 41598_2017_15829_MOESM1_ESM.pdf]

Supplementary Material: An algorithm for the beat-to-beat assessment of cardiac mechanics during sleep on Earth and in microgravity from the seismocardiogram.

Marco Di Rienzo, Emanuele Vaini, Prospero Lombardi

Dept. of Biomedical Technology, Fondazione Don Carlo Gnocchi, ONLUS, Milano, Italy

CONTENT OF THE DOCUMENT

Part I of this Supplementary Material provides details on the simulation analysis used for determining the maximal error in the PPV estimates computed in the main article.

Part II provides details on the ancillary analysis on the algorithm portability.

PART I - EVALUATION OF THE PPV ESTIMATION ERROR

As shown in the main article, the statistical evaluation of the algorithm performance led to PPVs close to 1 (or 100% when expressed in percent). It was reported that for high PPVs the estimation error can hardly be derived from analytical formulas (because of the asymmetric distribution caused by the right-hand limit to 1) and simulations should be used [1].

Thus, to evaluate the estimation error of our PPV measures we developed a specific simulation procedure. As test data set, simulating the real PPV, we considered an array of 23,000 boolean elements all initialized to TRUE. The length of the array approximates the number of heart beats in our recordings.

The simulation considered PPVs ranging from 0.9 to 0.999 with step 0.001. For any given PPV, an appropriate number of elements was set to FALSE (Number of FALSE=(1-PPV)*23,000) with a randomly selected position in the dataset array. For any given PPV, a million estimates were carried out. Per each estimate, 2300 elements of the array (corresponding to the 10% of the dataset, like in the evaluation of the algorithm) were randomly selected and the number of TRUE values observed in the sample was counted. The estimated PPV was computed as Number of TRUE/23000. Finally for every PPV, the mean, minimal and maximal value of the 1,000,000 PPV estimates were considered.

The results of the simulation are shown in the Table S1. For space reasons the results are reported with the original 0.001 step only for the PPVs ≥ 0.99, while results referring to lower PPVs are reported with a progressively larger step (0.002, 0.005, 0.01).

Table data are also plotted in Fig. S1, to facilitate the exploitation of this simulation. In the graph there are three curves: the blue continuous curve in the middle is the identity line between the simulated and the estimated PPVs, the blue squares on the left and the green triangles on the right represent the maximal and minimal estimated values reported in the table, respectively. The range of error for any given estimated PPV may be simply obtained by drawing a horizontal line at the desired level of the ordinate and by measuring the corresponding abscissa values at the points of intersection of the line with the min and max curves. Those values represents the minimal and maximal range of the error for the given estimated PPV.

It is worth noting that the range of the estimation error progressively decreases as the PPV tends to 1 and that the largest error may be observed on the left side of the identity line. This asymmetry may be expected because of the closeness with the unity boundary.

In the figure we also reported the estimation error for the minimal PPV we obtained in the evaluation of the algorithm (0.992, corresponding to the MO estimation on the on-ground recording). It appears that on the basis of the simulation, this estimation value corresponds to real PPV values ranging from 0.981 to 0.997. Thus the absolute maximal error is 0.992-0.981=0.011 corresponding to 1.1%. The PPV estimates for the remaining FPs were higher than 0.992 and thus their maximal estimation error is less than 1.1%.

PART II - ALGORITHM PORTABILITY

In the main article we described the details of the algorithm designed for the analysis of data recorded in a specific subject (the astronaut). This development demonstrated the feasibility of an automatic estimation of indexes of cardiac mechanics during sleep from SCG recordings taken in real-life scenarios very far from the traditional controlled condition of a laboratory setting. However, the positive results on the astronaut leaves virtually unaddressed the issue of the algorithm portability. In this frame, it might be conceived that the algorithm was so tightly tailored on the SCG waveform of the target subject to make it impossible its adaptation for the analysis of data from any other subject with reasonable efforts.

While the comprehensive exploration of the algorithm portability is out of the scopes of the present study and would require a separate investigation, in this ancillary analysis, we provide evidence that a tuned version of the algorithm - as obtained by the only change of some threshold values- may be employed to analyze data of different subjects.

SUBJECTS AND DATA COLLECTION - Two healthy male volunteers (age: 25 and 30 years) were recruited for this analysis. In each subject, ECG and SCG were recorded during sleep by a proprietary device, the SeisMote system, developed in our laboratory and functionally similar to the MagIC-Space system used for collecting data from the astronaut. The electronic module of SeisMote was the same used in the MagIC-Space, but, in the SeisMote system the ECG was detected by traditional gel electrodes (instead of the textile electrodes of the vest), and the SCG was acquired by an external accelerometer (instead of the accelerometer positioned inside the electronic module). During the data monitoring, the external accelerometer was placed on the lower part of the subject's sternum by a strip of adhesive tape. Similarly to the space experiments, data were sampled at 200Hz and locally stored on a memory card inside the electronic board. Structure and setup of the SeisMote system are shown in fig. S2, details of the device may be found in [2].

Each subject was instrumented and the recording was activated at around 6 p.m. in our laboratory. During the ongoing data monitoring, the subject went home, had dinner, spent the evening at home and then went to sleep. On the next morning, the subject stopped the monitoring at the wake up and dismounted the system. The times when the subject went to bed and woke up were annotated. Subsequently, data were downloaded from the system to a computer for the analysis. The first step of the elaboration was the extraction of the sleep data from the whole recording. The individual duration of the sleep data segment and the number of beats occurred at night are shown in table S2. Before being analyzed for the algorithm tuning, each sleep data segment, from now on termed "sleep recording", was pre-processed as done in the main analysis, thus the artifacts were manually removed from the ECG and the SCG was pass-band filtered.

THE TUNING WORKFLOW - The original algorithm keeps trace of any discarded beat and any missing FP, and annotates the algorithm step in which the failure occurred. The steps are: data segmentation, gross artifact removal, fine artifact removal, ICP identification, MC identification, AO identification, IRP identification, AC identification, MO identification, FP interpolation & re-sampling, congruency check. The number of discarded beats observed at each step of the algorithm in the on-ground recording of the astronaut was taken as a reference for the tuning. As detailed in the main article, each step includes a number of threshold tests. The tuning procedure was essentially based on a progressive refinement of some of the algorithm threshold values as obtained by a reiterated data analysis.

Since the quality of the new recordings was visually similar to the quality of the astronaut data, we assumed that at the end of the tuning procedure, the number of discarded beats in the new recording at each step of the algorithm should have been lower or close to the reference. On this premise, in the first iteration of the tuning procedure we analyzed the sleep recording with the algorithm in its original form (i.e. with the threshold values set for the astronaut). At the end of

this iteration, we checked the number of beats discarded in each step of the procedure, and identified the first step in which the number of rejected beats exceeded the reference. Then, by a visual inspection of some of the discarded beats, we could determine which threshold test failed among those in the step, and set a new threshold value. The whole recording was then re-analyzed by the updated version of the algorithm and the procedure was reiterated till the number of discarded beats was lower or close to the reference for all the steps of the algorithm.

At the end of the tuning, we also checked the precision of the FPs identification through the PPV. For the estimation we kept the same procedure and sample size (10% of the available data) we used in the main analysis. We planned that in case of a PPV <95% the tuning had to be further refined, but in both subjects this event did not occur.

RESULTS - The list of the threshold values we modified for adapting the algorithm to the analysis of the new sleep recordings is shown in Table S3. The table shows, for each subject, the algorithm step in which the change was made, the threshold test involved, and the new value of the threshold. The corresponding values set for the astronaut are also reported as reference.

In each subject the final tuning was reached after the change of two threshold values related to two different steps of the algorithm for subject 1 (the gross artifact rejection, and the IRP identification), and of three values related to three steps for subject 2 (the gross artifact rejection, the ICP identification and the IRP identification). The largest inter-subject variability in the setting was found in the size and position of the search window around Te used for the identification of the IRP anchor point.

The signal quality and the FPs incidence resulting from the analysis of the two recordings by the tuned versions of the algorithm are shown in Table S4. For each FP, the table also includes the corresponding PPV value, and the relevant astronaut data, taken from Table 2 and 3 of the main article, as reference.

The analyses obtained by the tuned procedures indicate that when compared with the reference, the new recordings are characterized by a similar signal quality and a similar incidence of the MC and AO fiducial points; while it was observed a higher incidence of the AC and MO fiducial points, and an even higher incidence of beats with all four FPs identified (>+25%).

For both subjects PPVs were always >99%. This means that the precision of the tuned algorithms to correctly identify the FPs is comparable with that of the original algorithm applied to the astronaut data.

CONCLUSIONS - The results of this ancillary analysis provide evidence that the proposed algorithm may be tuned for the analysis of data of subjects different from the astronaut, with the only change of some threshold value without requiring any change in the algorithm structure. Although obtained from an extremely limited dataset, this finding excludes the hypothesis that our original

algorithm was so tightly tailored on the SCG waveform of the astronaut to make it impossible the adaptation to any other subject with reasonable efforts. Obviously, the determination of the extent of the algorithm portability to a wider population and to different subgroups of subjects (classified by gender, age, body mass index, health status, etc.) is out of the scope of the present study and remains to be explored by a separate investigation.

A note on the workflow we used for the tuning. The design of the tuning procedure we adopted in this test was not optimized, thus it is possible that a better and faster tuning of the algorithm might be obtained by a more sophisticated and automated workflow.

As to the timing of the current tuning, the computation time of each iteration of the procedure took a couple of minutes, while the final determination of each new threshold value took 3-4 hours. However, it should be mentioned that most of the time spent for this ancillary analysis was not taken by the tuning procedure, but by our final verification of the correctness of the tuning obtained by the PPV estimation. Indeed, this task implied the visual check of 10% of the available data for each of the four FPs, for a total of (2,355+2,666)*4= 20,084 beats, and several days have been necessary for this validation.

References

[1] Pepe, MS. The statistical evaluation of medical tests for classification and prediction. Oxford University Press, (2003).

[2] Di Rienzo, M., Lombardi, P., Scurati, D., Vaini, E. *A New Technological Platform for the Multisite Assessment of 3D Seismocardiogram and Pulse Transit Time in Cardiac Patients*. In: Computing in Cardiology 2016; 43:781-784 (2016).

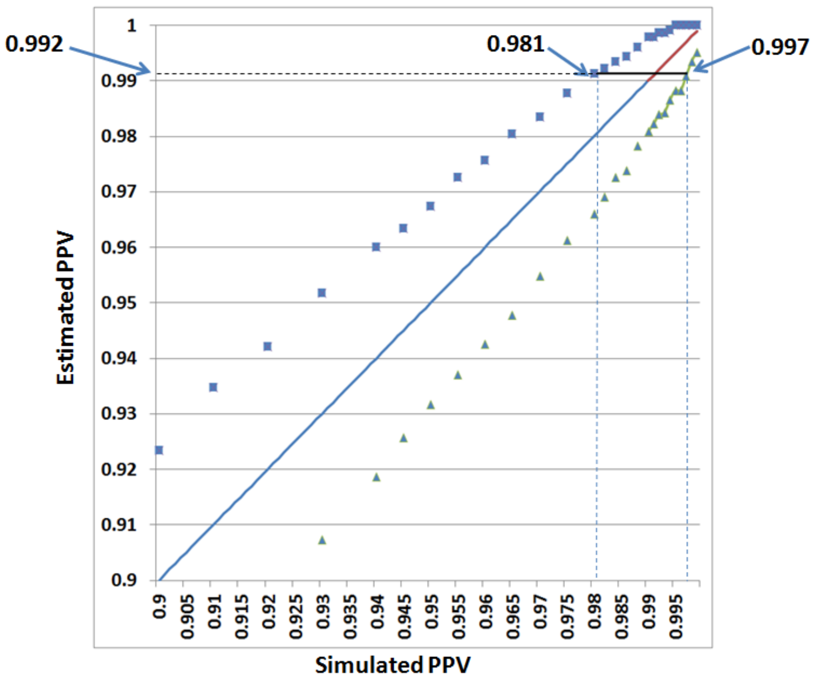

Figure S1 - Graphical representation of the simulation data reported in table A1. The continuous blue line is the identity line, the curve on the left (blue squares) and the curve on the right (green triangles) respectively corresponds to the Max estimated PPV and Min estimated PPV of the table. Computation of the maximal error in the PPV estimates of the algorithm performance is shown in the upper part of the figure. See text for details on how the estimation error may be obtained from the graph.

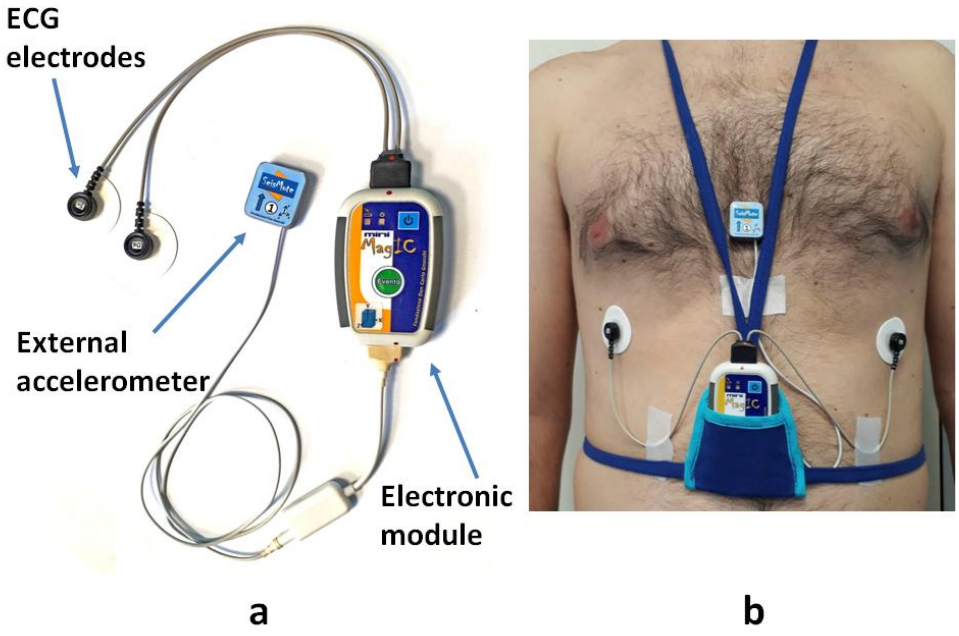

Figure S2 - The SeisMote system. Panel a: components of the system (see text for details). Panel b: Subject instrumented for the sleep recording.

Simulated PPV	Average estimated PPV	Min estimated PPV	Max estimated PPV
0.9	0.9001	0.8717	0.92348
0.91	0.91	0.8857	0.93478
0.92	0.92	0.8957	0.94217
0.93	0.93	0.9074	0.95174
0.94	0.94	0.9187	0.96
0.945	0.945	0.9257	0.96348
0.95	0.95	0.9317	0.96739
0.955	0.955	0.937	0.97261
0.96	0.96	0.9426	0.97565
0.965	0.965	0.9478	0.98043
0.97	0.97	0.9548	0.98348
0.975	0.975	0.9613	0.98783
0.98	0.98	0.9661	0.9913
0.982	0.982	0.9691	0.99217
0.984	0.984	0.9726	0.99348
0.986	0.986	0.9739	0.99435
0.988	0.988	0.9783	0.99609
0.99	0.99	0.9809	0.99783
0.991	0.991	0.9822	0.99783
0.992	0.992	0.9839	0.9987
0.993	0.993	0.9843	0.9987
0.994	0.994	0.9865	0.99913
0.995	0.995	0.9883	1
0.996	0.996	0.9883	1
0.997	0.997	0.9909	1
0.998	0.998	0.9935	1
0.999	0.999	0.9952	1

Table S1 - Results of the simulation. The first column contains the nominal PPV, the following columns respectively contain the average, the maximal and the minimal value of the 1,000,000 PPV estimates.

	Duration	No. beats
**Subject 1**	6h54m	23,550
**Subject 2**	7h04m	26,657

Table S2 - Details of the new sleep recordings. Individual duration and number of occurred beats.

Algorithm Step & Test	Reference	Subject 1	Subject 2
Gross artifact rejection (SCG max amplitude)	<50mg	≤70mg	≤60mg
ICP identification ($\lvert ICP_{Rdelay} - ICP_{Rdelay}^{ref} \rvert$)	≤ +30ms	No change	≤ +20ms
IRP identification (dimension of the search window around T_e for the IRP identification)	From -30 to +30 ms	From +25 to +90 ms	From -20 to +45 ms

Table S3 - Details of the algorithm tuning for the analysis of the new sleep recordings. For each subject, the algorithm steps in which the change was made, the threshold tests involved, and the new values of the threshold are shown. The corresponding values set for the analysis of the astronaut data are also reported as reference.

	Reference	***Subject 1***	***Subject 2***
SCG quality (artifact-free beats)	91.0	95.3	92.9
SCG fiducial points			
MC (*PPV*)	99.0 (*99.9*)	97.8 (*99.6*)	97.1 (*99.7*)
AO (*PPV*)	96.7 (*99.9*)	98.2 (*100*)	99.8 (*99.8*)
AC (*PPV*)	77.7 (*99.4*)	95.3 (*99.7*)	83.1 (*99.4*)
MO (*PPV*)	72.9 (*99.2*)	90.6 (*99.1*)	99.0 (*99.9*)
All 4 fiducial points	64.2<br>(13,716 beats)	86.6<br>(19,333 beats)	81.0<br>(20,037 beats)

Table S4 - Signal quality and FPs incidence, in percent, resulting from the analysis of the two recordings by the tuned versions of the algorithm. For each FP, it is also reported the corresponding PPV value, in percent, indicating the level of precision in the identification. The relevant astronaut data taken from Table 2 and 3 of the main article are also included as reference.
